# Analgesic Strategies for Urologic Videolaparoscopic or Robotic Surgery in the Context of an Enhanced Recovery after Surgery Protocol: A Prospective Study Comparing Erector Spinae Plane Block versus Transversus Abdominis Plane Block

**DOI:** 10.3390/jcm13020383

**Published:** 2024-01-10

**Authors:** Marco Micali, Giada Cucciolini, Giulia Bertoni, Michela Gandini, Marco Lattuada, Gregorio Santori, Carlo Introini, Francesco Corradi, Claudia Brusasco

**Affiliations:** 1 Anaesthesia and Intensive Care Unit, E.O. Ospedali Galliera, 16128 Genoa, Italy; michela.gandini@galliera.it (M.G.); marco.lattuada@galliera.it (M.L.); claudia.brusasco@gmail.com (C.B.); 2 Department of Surgical, Medical, Molecular Pathology and Critical Care Medicine, University of Pisa, 56126 Pisa, Italy; giada.cucciolini@phd.unipi.it (G.C.); francesco.corradi@unipi.it (F.C.); 3 Anaesthesia and Intensive Care Unit, NOA—Nuovo Ospedale Apuano, 54100 Massa, Italy; giuliabertoni88@gmail.com; 4 Department of Surgical Sciences and Integrated Diagnostics (DISC), University of Genoa, 16126 Genoa, Italy; gregorio.santori@unige.it; 5 Department of Abdominal Surgery, Urology Unit, E.O. Ospedali Galliera, 12128 Genoa, Italy; carlo.introini@galliera.it

**Keywords:** postoperative analgesia, plexus abdominal blocks, abdominal surgery, laparoscopic urologic surgery, robotic urologic surgery, post-operative nausea and vomiting, ERAS protocol, postoperative pain, opioid-free analgesia, opioid-sparing analgesia

## Abstract

Regional anesthesia in postoperative pain management has developed in recent years, especially with the advent of fascial plane blocks. This study aims to compare the ultrasound-guided bilateral erector spinae plane block (ESPB) versus the ultrasound-guided bilateral transversus abdominis plane block (TAPB) on postoperative analgesia after laparoscopic or robotic urologic surgery. This was a prospective observational study; 97 patients (ESPB-group) received bilateral ultrasound-guided ESPB with 20 mL of ropivacaine 0.375% plus 0.5 mcg/kg of dexmedetomidine in each side at the level of T7–T9 and 93 patients (TAPB-group) received bilateral ultrasound-guided TAPB with 20 mL ropivacaine 0.375% or 0.25%. The primary outcome was the postoperative numeric rating scale (NRS) pain score, which was significantly lower in the ESPB group on postoperative days 0, 1, 2, and 3 (*p* < 0.001) and, consequently, the number of patients requiring postoperative supplemental analgesic rescue therapies was significantly lower (*p* < 0.001). Concerning the secondary outcomes, consumption of ropivacaine was significantly lower in the group (*p* < 0.001) and the total amount of analgesic rescue doses was significantly lower in the ESPB-group than the TAPB-group in postoperative days from 2 to 4 (1 vs. 3, *p* > 0.001). Incidence of postoperative nausea and vomiting was higher in the TAPB group and no block-related complications were observed. Our data indicate that ESPB provides postoperative pain control at least as good as TAPB plus morphine, with less local anesthetic needed.

## 1. Introduction

Minimally invasive surgery associated with enhanced recovery after surgery (ERAS) programs is a standard of care in many hospitals around the world nowadays, as it has demonstrated a reduction in post-operative recovery time and complications, without affecting the oncological outcomes [1,2,3,4].

However, even if the pain is reduced by the surgical technique itself due to small keyhole incisions with limited tissue retraction and stretching of fascia and muscular fibers, residual pain arises from abdominal distension and peritoneal irritation due to pneumoperitoneum, diaphragm stretching, trocar incision, the abdominal wall extraction site, and internal organs [5].

In accordance with ERAS protocols, the anesthetic and analgesic techniques have been changing for years and nowadays, multimodal strategies for post-operative pain management have been identified in order to spare partially or totally the use of opioids and non-steroid anti-inflammatory drugs (NSAIDs) [6]. Opioids are in fact responsible for nausea, vomiting, or respiratory depression while NSAIDs increase the risk of postoperative acute kidney injury (PO-AKI), especially in urologic patients [6,7].

Among current analgesic strategies for laparoscopic or robotic-assisted urologic surgery, there are neuraxial analgesia, (i.e., epidural or spinal analgesia) or inter-fascial blockages, such as a transversus abdominis plane block (TAPB) or erector spinae plane block (ESPB) [5,6,8]. TAPB involves an injection of local anesthetic between the internal oblique and transverse muscles. This interfascial plane contains the intercostal, subcostal, iliohypogastric, and ilioinguinal nerves as well as branches from T9 to T12 intercostal nerves and from L1. These nerves give sensation to the anterior and lateral abdominal wall and the parietal peritoneum, providing only somatic and not visceral analgesia. This technique has become one of the most common fascial blocks performed for postoperative analgesia after abdominal surgeries and is considered easy and quick to perform. ESPB is a more recent technique, first described in 2016. It is an easy-to-perform and relatively safe technique, consisting of an ultrasound-guided local anesthetic injection in a plane between the erector spinae muscle and the underlying transverse process. It can be performed at various levels, both thoracic and lumbar, depending on the type of surgery. Several studies have compared the efficacy of these techniques and the superiority of one among others with controversial results [9,10]. Nevertheless, we still do not have a current recommendation for the use of one technique over the others and the choice still relies on the on-duty anesthetist.

The aim of the present study was to describe the analgesic ERAS protocols in use at our institution and to compare the analgesic efficacy of intraoperative TAPB versus ESPB for postoperative pain control in minimally invasive urologic surgeries through the NRS pain scale and the number of supplemental analgesic rescue therapies requested. The total local anesthetic requirement, reduction in opioids or NSAIDs consumption, and incidence of adverse effects or block-related complications will also be evaluated as secondary outcomes.

## 2. Materials and Methods

### 2.1. Study Design and Patients

This was an observational prospective study conducted at the Galliera Hospital of Genova from January 2022 to July 2023. All patients signed informed consent on personal data storage and the local ethics committee approved the study (7/2019 id: 4378, amendment 2). The study involved 190 consecutive patients undergoing any type of major elective urologic surgery by laparoscopic or robotic-assisted techniques. All patients followed a consolidated perioperative ERAS program as previously described [7]. The only exclusion criteria were the inability to perform an inter-fascial blockage due to infection at the injection site, known allergy or hypersensitivity to local anesthetics, or serious heart arrhythmia. In total, 93 patients were submitted to the TAPB analgesic protocol (TAPB-group) and 97 to the ESPB analgesic protocol (ESPB-group). The analgesic protocols for both groups have been previously defined in the ERAS protocol and patients were submitted to either one depending on the anesthesiologist’s skill and personal clinical decision (Table 1).

### 2.2. Technique

#### 2.2.1. ESPB 

**Figure 1:**
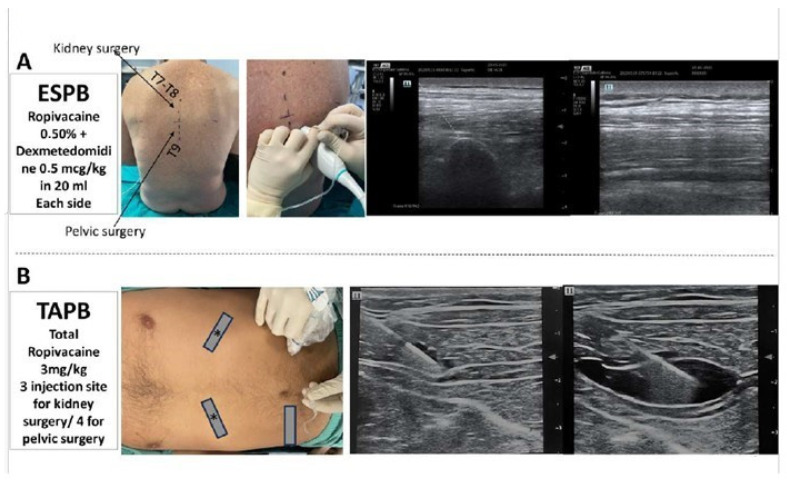
Panel (**A**): Erector spinae plane block (ESPB); from the left: level of puncture, in-plane technique, ultrasound visualization of transverse process, and ultrasound image of anesthetic spread. Panel (**B**): transversus abdominis plane block (TAPB); from the left: position of the probe for four or three puncture sites (box with star, subcostal approach; simple box, lateral approach) with in-plane technique, an ultrasound image of needle puncture, and anesthetic spread through the fascia delimited by the internal oblique and the abdominal transverse muscle.

Patients were taken to a preanesthetic room 1 h before surgery. After placement of an 18–16 G peripheral intravenous cannula, patients were pre-medicated with midazolam 1–3 mg i.v. and monitored by pulse oximetry, electrocardiography, and non-invasive arterial pressure measurement. Patients were first positioned in a sitting position and an ultrasound evaluation was performed to identify the transverse process of the vertebrae corresponding to the desired level and the erector spinae muscle. ESPB was performed between T7 and T9 transverse processes, preferring T7–T8 for kidney surgery and T9 for pelvic surgery. A linear probe of 10 MHz was used for ultrasound evaluation and a convex probe with a musculoskeletal setting (Mindray TE7 ultrasound device, Shenzhen Mindray Bio-Medical Electronics Co., Shenzen, China) was used only if a deeper view was required by high BMI or particular physical conformation. Once identified, the transverse process between T7 and T9, a 22 G × 88 mm needle (SonoTAP, PAJUNK GmbH, Medizin Technologie, Wessling, Germany) was inserted after disinfection of the puncture site, using an in-plane technique with a 30° angle in a cranio–caudal direction; the needle was advanced through the muscle to hit the transverse process. After a negative aspiration test, ropivacaine 0.375% or 0.5% mixed with normal saline for 20 mL plus dexmedetomidine 0.5 mcg/kg was administered. During injection, the spread of the local anesthetic mixture was monitored in real time and the correct execution of the block was evidenced by the separation of the erector spinae muscle from the transverse process. The same procedure was repeated on the contralateral side (Figure 1A).

#### 2.2.2. TAPB 

TAPB was performed after induction of anesthesia. For pelvic surgery, TAPB was performed through four injection sites (two inferolateral and two subcostal sites bilaterally). For kidney surgery, due to the monolateral position of the trocar incisions, TAPB was performed through three injection sites (one inferolateral and two bilateral subcostal sites). The ropivacaine total dose was calculated depending on the number of injection sites and patient’s weight (total amount of ropivacaine 3 mg/kg divided into 3 or 4 injection sites). In the supine position, a linear 10-mHz transducer (Mindray TE7 ultrasound device, Shenzhen Mindray Bio-Medical Electronics Co., Shenzhen, China) was placed laterally on the abdominal wall between the lower costal edge and the iliac crest to identify the three abdominal wall muscles (external oblique, internal oblique, and transverse abdominis). After disinfection of the puncture site, a 22 G × 88 mm needle (SonoTAP, PAJUNK GmbH, Medizin Technologie, Wessling, Germany) was inserted latero-medially with an in-plane technique until it reached the fascia delimited by the internal oblique and the abdominal transverse muscle. (Figure 1B) After a negative aspiration test, ropivacaine 0.375% or 0.25% mixed with normal saline for 20 mL was injected. During injection, the spread of the local anesthetic mixture was monitored in real-time and the correct execution of the block was evidenced by the separation of the muscles.

#### 2.2.3. Anesthesia Management 

General anesthesia was induced with propofol (1.5–2 mg/kg), remifentanil at 0.15 mcg/kg/min, and rocuronium (1 mg/kg). Anesthesia was maintained with sevoflurane (MAC 0.5–0.8) and remifentanil infusion (0.005–0.2 mcg/kg/min) keeping the bispectral index value (BIS) between 50 and 60. A deep neuromuscular blockade was maintained throughout the surgery with a train-of-four (TOF) = 0 and a post-tetanic count (PTC) < 4. All patients were managed with an individualized goal-directed fluid therapy. Pressure-controlled ventilation with volume guaranteed was used for intraoperative mechanical ventilation. The tidal volume was set at 7–8 mL/kg and the respiratory rate was adjusted to maintain 30–35 mmHg end-tidal CO_2_. All patients had a pneumoperitoneum pressure of 10 mmHg, using the AirSeal Intelligent Flow System^®^ (ConMed, Utica, NY, USA) or Lexion System^®^ (Lexion Medical, St Paul, MN, USA) throughout the surgery procedure, either laparoscopically or in a robotic-assisted manner. Patients undergoing radical prostatectomy were positioned at 20° Trendelenburg for laparoscopic or 24° for robotic-assisted technique. Patients undergoing nephrectomy were placed in a lumbotomy position. Before awakening from anesthesia, all patients received intravenous paracetamol 1 gr. NSAIDs, namely, ketoprofen 30 mg or ibuprofen 600 mg, which were administered only to patients with an estimated glomerular filtration rate (eGFR) >60 mL/min/1.73 m^2^ otherwise no NSAIDs were administered. The TAPB-group also received morphine 5 mg while the ESPB-group did not receive any opioids. At the end of the procedure, patients were extubated by reversing rocuronium with 2–4 mg/kg sugammadex. Patients were monitored for 60 min in the recovery room and discharged when the modified Aldrete score system was >8.

#### 2.2.4. Analgesia Protocol and Rescue Analgesia

For postoperative analgesia (Table 1), patients received paracetamol 1 g every 8 h on PostOperative Day (POD)-0 and 1. From POD-2, no fixed analgesic therapy was foreseen but it was set up only as needed, with a first rescue therapy based on paracetamol and a second rescue therapy based on an NSAID (Ketorolac 30 mg). The second rescue therapy with an NSAID was excluded for patients at high risk of PO-AKI or with eGFR < 60 mL/min/1.73 m^2^ and in these cases, the second rescue dose was Morphine 3 mg s.c. Pain was evaluated by an 11-point numerical rating scale (NRS), with 0 indicating “no pain” and 10 indicating “the worst pain ever possible”; nurses asked patients to score pain three times a day, every 8 h. First rescue analgesia was administered when NRS was >4. If a first rescue dose was not sufficient and NRS was still >4, a second rescue analgesia was administered at the clinician’s discretion. Postoperative nausea and vomiting were evaluated by a verbal descriptive scale.

### 2.3. Outcomes

The primary outcome was to compare the postoperative mean and maximum NRS per day between groups from POD0 to POD4. Secondary outcomes included postoperative consumption of rescue therapies, the number of patients requiring postoperative supplemental analgesic rescue therapies, nausea and vomiting events, incidence of block-related complications (bleeding, infection, pneumothorax, and perforation), or other side effects (nausea, vomiting, bradycardia, and hypotension) and total local anesthetic consumption. 

### 2.4. Statistical Analyses

Assuming a difference in the proportion of postoperative complications between the two groups = 0.15 (effect size for proportions = 0.502), a minimum sample size of 63 patients for each group was required to obtain a statistical power = 0.8 (alpha = 0.05 with two-sided alternative hypothesis). Categorical data are presented as numbers and percentages and continuous data are presented as mean and standard deviation (SD). Categorical data were compared by Pearson’s χ^2^ test with Yates correction or Fisher’s exact test, when appropriate. Propensity score matching was performed to evaluate potential unbalanced variables between ESPB and TAPB groups. An inverse probability of treatment weighting (IPTW) with a propensity plot was also evaluated. A standardized mean difference (SMD) was calculated for each comparison. Adjusted logistic regression (LR) models were carried out to detect putative predictors for rescue dose administration. Univariate LR models that reached statistical significance were entered in a multivariate model. For each LR model, the odds ratio with 95% confidence interval (CI) was returned. For the multivariate LR model, we calculated the overall accuracy, specificity, sensitivity, and area under the ROC curve (AUC), by providing the corresponding cut-off plot. A two-tailed *p*-value < 0.05 was assumed for statistical significance. Statistical analyses were performed using the SPSS software (version 27.0; SPSS, Chicago, IL, USA) and R environment (version 4.2.3, R Foundation for Statistical Computing, Vienna, Austria).

## 3. Results

**Figure 2:**
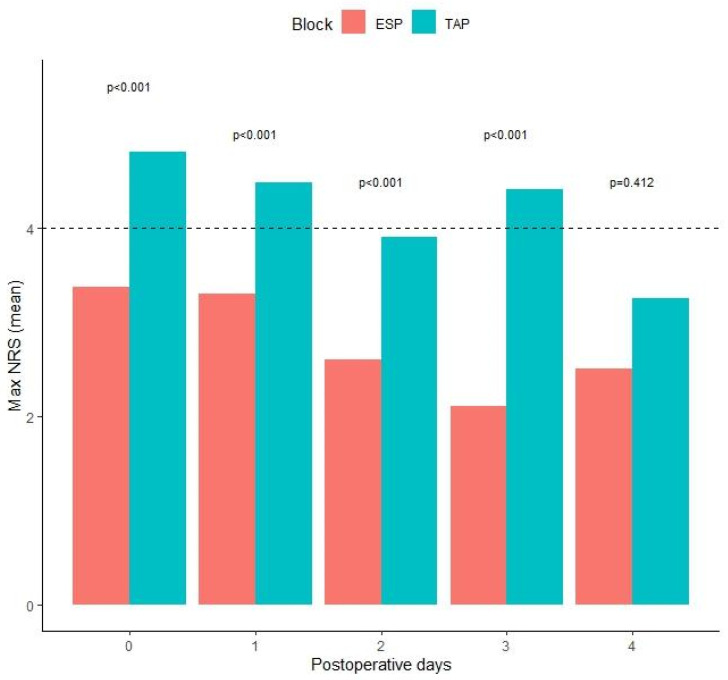
Trend of changes of the postoperative numeric rating scale (NRS) in both groups from postoperative day 0 to 4. ESPB: erector spinae plane block. TAPB: transversus abdominis plane block.

**Figure 3:**
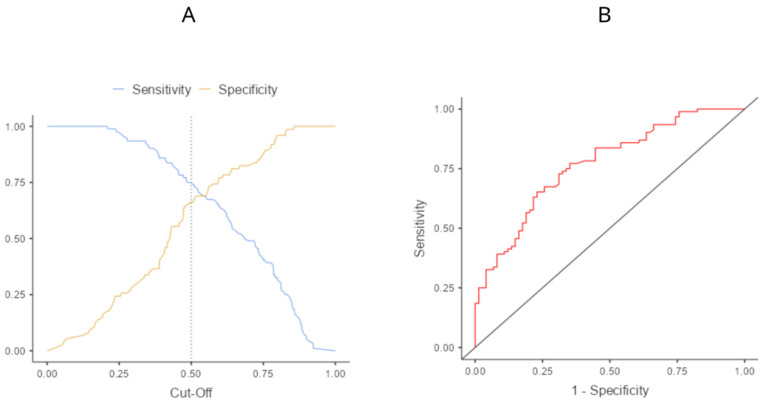
Cut-off plot (**A**) and ROC curve (**B**) of the multivariate logistic regression model with rescue dose administration as dependent variable.

The two groups were comparable regarding age, sex, BMI, ASA physical status, type and duration of surgery, and duration of anesthesia, while a statistically significant difference was reported in conserving pneumoperitoneum pressures and intraoperative fluids administration (Table 2). Maximum NRS was significantly lower in either POD0, POD1, POD2, and POD3 in the ESPB group than in the TAPB group (*p* < 0.001, Table 3 and Figure 2). Moreover, NRS mean values in all PODs were lower than four for the ESPB group while they were higher than four in the POD0, POD1, and POD3 TAPB group. However, in POD2, POD3, and POD4, the number of patients with no pain was significantly higher in the ESPB group (Table 4). In the ESPB group, there was a lower number of patients who experienced pain during all postoperative days with a statistically significant difference on POD2, POD3, and POD4 (*p* = 0.021, *p* = 0.023, and *p* = 0.004, respectively). Moreover, the number of patients who experienced NRS > 4 was statistically lower in the ESPB compared to the TAPB group in POD0, POD1, POD2, and POD3 (*p* = 0.002, *p* < 0.001, *p* < 0.001, and *p* < 0.001, respectively) with a consequent significant lower number of patients requiring rescue doses in the ESPB group (Table 5). In POD4, pain was rarely present because of the minimally invasive surgical approach; thus, no significance was reached between groups. A significantly lower amount of ropivacaine was administered to patients of ESBP than the TAPB group (170 ± 25 vs. 305 ± 25, *p* < 0.001). The number of analgesic rescue doses was insignificantly different between groups in POD0 and POD1 but significantly lower in ESPB than TAPB group in POD2 and POD4 (1 vs. 3, *p* < 0.001). Comparing type of rescue therapies administered, the ESPB group required significantly less paracetamol (*p* = 0.005), NSAID (*p* < 0.001), and morphine (*p* = 0.033). A significantly lower occurrence of postoperative nausea and vomiting (PONV) was reported by ESPB than in TAPB patients (18 vs. 4, *p* < 0.001). No block-related complications were observed in any patient in the two groups. Only three vasovagal events were reported in the ESPB group during the plexus procedure but none of them was so relevant to prevent the block from being completed. Propensity score matching and IPTW returned a potential imbalance for surgical technique. Thus, each LR model with rescue dose administration as a dependent variable was adjusted for the surgical technique. Univariate LR returned a statistical significance for age (*p* = 0.027) and fascial plane block (*p* = 0.003) (Table 6). The multivariate model confirmed the statistical significance as being the type of inter-fascial analgesia and the strongest independent variable providing a unique statistically significant contribution to the model controlled for all other factors. The model provided an overall accuracy = 0.705, a specificity = 0.662, a sensitivity = 0.739, and AUC = 0.768 (Figure 3).

## 4. Discussion

This prospective observational study evaluated the efficacy of intraoperative TAPB versus ESPB for postoperative pain control, in addition to the development of complications due to the inter-fascial blocks, opioids or NSAIDs consumption, and need of rescue analgesic doses. The main findings were that an analgesic strategy with ESPB (1) achieved a lower total number of patients experiencing pain in all postoperative days and lower values of NRS in those patients experiencing postoperative pain, (2) was obtained in a totally opioid-free mode and with lower ropivacaine doses, and (3) required less postoperative analgesic rescue therapies (paracetamol, NSAIDS, and morphine), thus reducing postoperative nausea and vomiting.

Several studies have demonstrated the effectiveness of ESPB in providing analgesia for thoracic, abdominal, and lumbar surgeries, depending on where it is performed [11]. Indeed, ESPB at low thoracic levels (T8–T12) effectively affects the abdominal dermatomes involved in major urological surgery [12]. Post-mortem studies have shown that 15–20 mL of local anesthetics are able to produce a craniocaudal spread from the injection point to 4–5 transverse processes above and below [13]. Moreover, a simultaneous spread of local anesthetic to craniocaudal and paravertebral areas affects dorsoventral branches of spinal nerves and sympathetic ganglia, thus achieving either somatic or visceral sensory blockade. This allows the use of opioids both intra- and post-operatively to be reduced substantially, thus limiting adverse effects [14]. Our results confirm those of previous studies demonstrating a high efficacy of ESPB in an opioid-free strategy and extending ESPB efficacy to minimally invasive urologic surgeries. In fact, up to date, only a few experiences on ESPB in urologic surgery have been reported for percutaneous nephrolithotomy [15,16], radical prostatectomy either laparoscopic or open [12,17], and laparoscopic nephrectomies [18,19], mostly comparing ESPB to epidural or completely intravenous analgesia.

TAPB provides a blockade of the cutaneous nerves of anterior and lateral abdominal walls from T6 to T12 [8], affecting somatic but not visceral components of pain. For this reason, low opioid doses are required upon awakening from surgery to cover the immediate postoperative period. However, even if in our study, we demonstrated TAPB to be inferior to ESPB; an analgesic strategy with TAPB achieves great benefits compared to standard analgesic treatments in the first 24–48 h after surgery in terms of pain scores and overall opioid consumption. In fact, TAPB has been described to be effective in analgesic pain control with a reduction in opioid consumption even in urologic surgery with good results in either radical robotic-assisted prostatectomies [20], laparoscopic nephrectomies [21], and percutaneous nephrolithotomy [22]. 

Concerning the complexity of postoperative pain from an etiopathological point of view and its subjective nature, in our study, we performed a logistic regression analysis aimed at defining the role of any confounding or pain-influencing factors that could have played an important role on patients’ postoperative pain. Among these, we evaluated either demographic factors such as age, sex, body weight, smoke, and degree of associated comorbidities assessed by ASA score as well as either intraoperative factors including the pneumoperitoneum pressures applied, amount of liquids infused, surgical technique (videolaparoscopy or robotic), and duration of surgery or the site of surgery distinguishing it into two quadrants (pelvic surgery, i.e., prostatectomies or bladder surgery and upper abdomen, i.e., kidney surgery). From this analysis, the strongest independent contribution to postoperative pain was provided by the different types of analgesic protocols performed. 

From a technical point of view, in our study, we chose a multiple-site TAPB technique, using both subcostal and lateral approaches, with different numbers of injection sites, depending on the positioning of trocar edges for the different types of surgery. Four injection sites were chosen for all pelvic procedures, while for nephrectomies, we opted for three injection sites: two subcostal and one lateral on the intervention side [8]. The latter enabled us to inject concentrations of ropivacaine in the organ extraction area requiring a surgical enlargement of the trocar edge [21] higher than in the two subcostal bilateral sites, aimed at attenuating pain from pneumoperitoneum overdistension. The total volume for each site was always 20 mL as in numerous previous studies and the ropivacaine total dose was determined as the maximal safe dose. On the contrary, the two injection sites required for ESPB have demonstrated a significant reduction in the total dose of local anesthetics, with a consequent significant reduction in the risk of systemic toxicity [23].

Another technical aspect to be underlined is that TAPB was performed during general anesthesia before starting surgical procedures, to avoid patient discomfort or interference. On the contrary, ESPB required collaboration by the patient being in a sitting position. This was likely the reason for three vasovagal episodes in our ESPB group, which were quickly resolved by placing the patient in a supine position and administering a bolus of epinephrine. Neither group had side effects during intraoperative or postoperative periods.

We decided to use dexmedetomidine in ESPB as an adjuvant to prolong our inter-fascial block due to a reduction in neuronal activity, reduction in acute local anesthetic-induced perineurial inflammation, and local vasoconstriction able to delay local anesthetic absorption [24,25,26]. Moreover, by adding dexmedetomidine we also took advantage of some systemic effects such as mild sedative, anti-anxiety, and hypnotic [27,28]. The optimal dose has not yet been determined. In our ERAS protocol, we added dexmedetomidine to ESPB at a total dose of 1 μg/kg according to previous studies [27,29], which provided mild sedative and relaxing effects, facilitating patients’ management both on entering the operating room and awakening from anesthesia [29] and a long-lasting block up to 48–60 h [27]. This enabled us to avoid opioid administration, as rescue doses on POD1 and POD2, with a significant reduction in postoperative PONV. Furthermore, we were able to substantially reduce the use of NSAIDs, especially in POD2, with a lower risk of postoperative acute kidney injury and gastric ulcers. Finally, at the dosage used in this study, no serious side effects occurred in terms of hemodynamic instability or bradycardia.

This study has several limitations. First, because of its observational nature, the results need to be confirmed in further randomised controlled studies. Second, NRS pain scores were collected by nurses during nursing times. The maximum NRS and mean NRS per day were taken into account for analysis. No different assessments were taken at rest and physical activity, although per our ERAS protocol, all patients in POD1 are already out of bed and walking. Third, we did not assess block success and lasting by evaluation of dermatomal sensory loss using pinprick test or cold alcohol swab, which might have contributed to better define efficacy of our sensory blockades. Fourth, NRS > 4 was per protocol the cut-off value determining the supplemental administration of analgesic rescue therapies and all statistical analysis of the study was performed on this clinical cut-off value deriving from usual clinical practice. Fifth, the two analgesic strategies were identical regarding postoperative analgesic administrations but different intraoperative strategies were adopted, mainly as no adjuvants of local anesthetic were used in the TAPB.

## 5. Conclusions

An opioid-free analgesic strategy with ESPB plus dexmedetomidine can reduce the need of rescue analgesic drugs and effectively controls postoperative pain in minimally invasive major urologic surgery. Moreover, ESPB requires lower dosage of local anesthetic than TAPB, thus reducing the risk of systemic toxicity. Therefore, ESPB may be a good analgesic strategy in the context of ERAS programs to avoid side effects of opioids and NSAIDs that may slow down post-operative recovery.

## Figures and Tables

**Table 1:** ERAS analgesic protocols.

**Intraoperative analgesia strategies**	TAPB + Morphine 5 mg + Paracetamol 1 gr + Ketorolac 30 mg/Ibuprofen 600 mg	
	ESPB + Paracetamol 1 gr + Ketorolac 30 mg/Ibuprofen 600 mg	
**Post-operative analgesia strategy**	POD 0–1	Paracetamol 1 gr × 3/die Ketorolac 30 mg if NRS > 4 (If NSAIDs forbidden, Morphine 3 mg s.c.)
	POD 2 until discharge	Paracetamol 1 gr if NRS > 4 Ketorolac 30 mg if paracetamol not effective (If NSAIDs forbidden, Morphine 3 mg s.c.)

ERAS—enhanced recovery after surgery, TAPB—transverse abdominis plane block, ESPB—erector spinae plane block, POD—postoperative day, NRS—numerical rating scale; s.c.—subcutaneous, NSAIDs—non-steroid anti-inflammatory drugs.

**Table 2:** Demographic data and baseline characteristics.

	TAPB-Group (n. 93)	ESPB-Group (n. 97)	*p* Value	SMD
Age, yr	64 ± 13	66 ± 12	0.174	0.198
Sex, m/f n	77/16	87/10	0.207	0.201
BMI, kg/m^2^	26 ± 4	25 ± 4	0.646	0.067
ASA class				
I	14	5		
II	68	81		
III	11	11		
Type of surgery, n (%)				
Partial nephrectomy	17 (18)	18 (19)		
Radical nephrectomy	13 (14)	13 (13)		
Radical prostatectomy	57 (62)	58 (60)		
Adrenalectomy	2 (2)	1 (1)		
other	4 (4)	7 (7)		
Intravenous intraoperative fluids, mL	1418 + 416	1232 + 375	**0.004**	**0.538**
Pneumoperitoneum pressure, mmHg	11 + 2	10 + 1	**<0.001**	**0.096**
VLS/robotic surgery, n	53/41	61/36	0.104	1.542
Duration of surgery, min	132 ± 55	150 ± 51	0.121	0.340
Duration of anesthesia, min	173 ± 53	177 ± 50	0.592	0.078

BMI—body mass index; VLS—video-laparoscopic surgery; ASA—American Society of Anesthesiology. Mean ± standard deviation. SMD—standardized mean difference.

**Table 3:** Comparison of the maximum NRS between groups.

	Maximum NRS (Mean ± SD)		
POD	TAPB-Group	ESPB-Group	*p* Value
0	4.81 + 1.95	3.37 + 1.65	**<0.001**
1	4.48 + 1.84	3.30 + 1.58	**<0.001**
2	3.89 + 1.88	2.60 + 1.03	**<0.001**
3	4.41 + 2.01	2.11 + 1.24	**<0.001**
4	3.25 + 1.57	2.50 + 1.91	0.412

POD—postoperative day; NRS—numerical rating scale.

**Table 4:** Comparison of the number of patients with no pain or with NRS ≤ 4 or NRS > 4 between groups.

	POD	TAPB-Group (n. 93)	ESPB-Group (n. 97)	*p* Value
No pain, NRS = 0	POD0	36	45	0.307
	POD1	22	24	0.867
	POD2	36	54	0.021
	POD3	61	78	0.023
	POD4	77	93	0.004
NRS > 4	POD0	31	13	0.002
	POD1	36	15	<0.001
	POD2	22	3	<0.001
	POD3	17	1	<0.001
	POD4	3	1	0.295

POD postoperative day; NRS numerical rating scale.

**Table 5:** Comparison of the quality of analgesia between groups and outcomes.

	TAPB-Group (n. 93)	ESPB-Group (n. 97)	*p* Value
Total ropivacaine consumption, mg	305 ± 60	170 ± 25	**<0.001**
Total patients requiring a rescue dose, n	55	25	<0.001
Total rescue dose requested, n	3 (2–5)	1 (0–2)	**<0.001**
Total NSAIDs consumption as rescue in POD1, n	40	26	0.092
Total morphine consumption as rescue in POD1, n	2	0	0.502
Total paracetamol consumption as rescue in POD2 and over, n	12	5	**0.005**
Total NSAIDs consumption as rescue in POD2 and over, n	29	6	**<0.001**
Total morphine consumption as rescue in POD2 and over, n	6	0	**0.033**
Time to first rescue analgesia, day	1 (0–1)	1 (0–1)	0.195
Intraoperative complications, n			
- bradycardia	0	0	0.999
- hypotension	0	0	0.999
Postoperative complications, n			
- PONV	18	4	**0.001**
- block related complications	0	0	0.999
Length of stay, n	4 (3–6)	4 (3–5)	0.401

NSAIDs—non-steroid anti-inflammatory drugs; PONV—postoperative nausea and vomiting; POD postoperative day. Mean ± standard deviation.

**Table 6:** Logistic regression analysis with rescue dose administration as dependent variable.

	Univariate		Multivariate	
Variables	OR (95% CI)	*p* Value	OR (95% CI)	*p* Value
Age	1.030 (1.003–1.057)	0.027	1.035 (1.003–1.069)	0.031
Sex	2.217 (0.922–5.332)	0.075		
BMI	1.049 (0.966–1.139)	0.253		
Smoke	0.942 (0.386–2.299)	0.895		
ASA class	2.038 (0.734–5.659)	0.172		
Type of surgery (upper/lower abdominal quadrant)	1.471 (0.794–2.725)	0.221		
TAPB/ESPB	3.179 (1.498–6.749)	0.003	2.703 (1.121–6.518)	0.027
Ropivacaine dose	0.998 (0.994–1.002)	0.318		
Intravenous intraoperative fluids	1.000 (0.999–1.001)	0.957		
Pneumoperitoneum pressure	1.020 (0.842–1.236)	0.840		
VLS/robotic surgery	0.871 (0.137–1.275)	0.125		
Duration of surgery	1.030 (0.997–1.009)	0.387		
Duration of anesthesia	1.001 (0.995–1.007)	0.885		

TAPB—tansversus abdominis plane block; ESPB—erector spinae plane block; BMI—body mass index; ASA—American Society of Anesthesiology; VLS—videolaparoscopic surgery.

## Data Availability

Data were not inserted in publicly archived datasets but are available anonymized for research purposes upon request to the corresponding author.
